# Spermatogenesis after Transplantation of Adipose Tissue-Derived Stem Cells in Azoospermic Guinea Pigs: A Histological and Histomorphometric Study

**DOI:** 10.22086/gmj.v0i0.1000

**Published:** 2018-07-13

**Authors:** Mehrdokht Hajihoseini, Davood Mehrabani, Akbar Vahdati, Seyed Ebrahim Hosseini, Amin Tamadon, Mehdi Dianatpour, Farhad Rahmanifar

**Affiliations:** ^1^Department of Biology, Fars Science and Research Branch, Islamic Azad University, Fars, Iran; ^2^Department of Biology, Shiraz Branch, Islamic Azad University, Shiraz, Iran; ^3^Stem Cells Technology Research Center, Shiraz University of Medical Sciences, Shiraz, Iran; ^4^The Persian Gulf Marine Biotechnology Research Center, The Persian Gulf Biomedical Sciences Research Institute, Bushehr University of Medical Sciences, Bushehr, Iran; ^5^Department of Medical Genetics, School of Medicine, Shiraz University of Medical Sciences, Shiraz, Iran; ^6^Department of Basic Sciences, School of Veterinary Medicine, Shiraz University, Shiraz, Iran

**Keywords:** Adipose Tissue, Stem Cell, Azoospermia, Infertility, Busulfan, Guinea Pig

## Abstract

**Background:**

The purpose of this research was to determine histomorphometric changes in busulfan-induced azoospermia after transplantation of Adipose Tissue-Derived Stem Cells (AdSCs) in guinea pig. AdSCs were isolated from adipose tissue around the testes of guinea pigs and characterized for mesenchymal properties.

**Materials and Methods:**

Guinea pigs were allocated into three groups, including the control group without any intervention. To induce azoospermia, groups 2 and 3 received a dose of 40 mg/kg of busulfan with 21 days interval. Group 3 received 1×106 AdSCs in their seminiferous tubules of left testes, 35 days following last busulfan injection, while right testis in the group was considered for comparison as controls. Sixty days following transplantation of cell, histomorphometric and histopathologic changes of the experiments were assessed.

**Results:**

After AdSCs’ transplantation, normal spermatogenesis appearance was noticed compared to busulfan-induced azoospermia and AdSCs recovered spermatogenesis, and our findings can be added to the literature in treating azoospermic infertilities.

**Conclusion:**

The transplanted AdSCs could induce production of germinal cells using testicular seminiferous tubules and were an effective source in treating azoospermia

## Introduction

Infertility is still a crucial social issue in many nations, while male infertility was demonstrated to be responsible for half of infertility cases [1] . Azoospermia is defined as lack of spermatozoa in the ejaculation without connoting underlying prominent etiology, influencing approximately 1% of males and 10-15% of childless men [2, 3]. It is classified into two types of non-obstructive or obstructive azoospermia, each with highly variable treatments and etiologies [4].

Obstructive azoospermia compromises 40% of azoospermic patients, commonly followed by preserving endocrine activity and normal exocrine, and natural spermatogenesis in the testis [5]. Obstructive azoospermia is due to physical blockage in the excurrent ductal system of males and may occur everywhere between the ejaculatory ducts and the rete testis [6]. Non-obstructive azoospermia affects approximately 60% of males with azoospermic, while abnormal testicular growth or toxic exposures are common causes of the disease [7]. Non-obstructive azoospermia includes primary, secondary and incomplete obscure failure of testicles [4]. In approximately two third of the men, azoospermia is related with incurable testicular disorders leading to failure of spermatogenesis, known as the most critical male infertility [3]. Azoospermia is confirmed according to examination of multiple semen samples as a transient azoospermia secondary to toxic, environmental, and infectious or iatrogenic conditions [8, 9]. Different treatments have been introduced for infertility of men involving micro-dissection testicular sperm extraction (MD-TESE) and *in-vitro* fertilization, particularly together with intra-cytoplasmic sperm injection (ICSI) [10].

The clinical management of males with azoospermia searching fertility is still a challenge for different specialists such as urologists, andrologists, and reproductive medicine specialists [11]. However, there is no influential medical therapy for patients with lack of mature sperm. Nowadays, stem cells are introduced for curing infertility of males caused by abnormalities in differentiation and proliferation of germ cells [12]. Transplantation of cell has been successfully used in various species with azoospermia [13, 14].

Mesenchymal stem cells (MSCs) possess similar features, including favorable proliferative capability, self-renewal, and differentiation potential [15]. These cells were separated from bone marrow [16], menstrual blood [17],

adipose tissue [18], and dental pulp [19]. They possess multilineage properties and can differentiate to osteoblasts [18], adipocytes [20] and neuronal-like cells [21]. MSCs are successfully used in treating azoospermia in animal models [14, 22, 23]. As adipose tissue is easily accessible and is a major source of MSCs making it a good candidate in regenerative medicine. The adipose tissue MSCs (AdSCs) are ubiquitous and easily provided in large numbers with slight donor site injuries or discomforting of patient, making them an appropriate research tool and therapy [24]. Thus, this investigation assesses histomorphometric and histological changes in guinea pig following AdSCs transplantation in busulfan-induced azoospermia.

## Materials and Methods

### Animals

Twelve outbred Dunkin-Hartley male guinea pigs (500-550 g) were purchased from the Comparative and Experimental Medicine Center, Shiraz University of Medical Sciences, Shiraz, Iran. They were kept in polypropylene cages with a 12-h lightning daily (7.00 a.m. to 7.00 p.m.) at 20-22°C and could freely have food and water. This research was approved in the Ethical Committee of Islamic Azad University. All experiments were performed based on the rules and regulations of Shiraz University of Medical Sciences for working on laboratory animals. The guinea pigs were allocated into three equal groups. The control was without any intervention. To induce azoospermia in groups 2 and 3, a dose of 40 mg/kg of busulfan (Busilvex®, Pierre Fabre Medicament, Boulogne, France) was injected intraperitoneally with 21 days interval as described before [25]. Group 3 received 1×10^6^AdSCs, 35 days after last injection of busulfan into seminiferous tubules of left testes.

### Isolation of Adipose Tissue-Derived Stem Cells (AdSCs)

AdSCs were provided using adipose tissue surrounding the testes while the guinea pigs were euthanized. Under sterile conditions, the isolated tissue was placed on ice to the stem cell laboratory. Briefly, adipose tissue was minced into tiny parts and was treated and shaken at 37°C for 40 min with 0.2% collagenase type II (Gibco, U.S.A.). The resultant was centrifuged (1500 ×g, 5 min) and filtered, and the precipitate was re-suspended in the medium of 5 mL Dulbecco’s Modified Eagles Medium (DMEM; Gibco, U.S.A.). The suspension was transferred into culture flasks containing 88% DMEM, 10% FBS (fetal bovine serum; Gibco, U.S.A.), and 1% penicillin and 1% streptomycin (Sigma, U.S.A.), and transferred into an incubator containing 5% CO2 at 37°C and saturated humidity. After 3 days, the medium was changed, and to wash the plate, PBS was used. Then, a DMEM having 10% FBS, 2 mM L-glutamine (Invitrogen, Netherlands) and 1% penicillin and 1% streptomycin were added and kept in the CO2 incubator. The cells were sub-cultured until passage 4.

### Cryopreservation of AdSCs

The isolated cells were cryopreserved for further cell transplantation. Therefore, for 3 to 4 min, the AdSCs’ confluent flasks in passage 4 were cured with 0.25% trypsin (Gibco, U.S.A.). The enzyme was inactivated using identical content of DMEM.

Centrifuging the cell suspension was conducted for 5 min at 1500 rpm. Then, to suspend the precipitate at 2×10^6^ viable cells/ml density, 50% DMEM media, 40% FBS, and 10% dimethyl sulfoxide (DMSO; MP Bio) were applied. The suspension was aliquoted to cryovials of sterile plastic labels.

The cryovials were kept at -20°C for 1 h, and then transferred to -70°C for 24 h, and at last, they were stored into liquid nitrogen for a long time. Whenever AdSCs were required, they were removed from nitrogen tank and transferred into a 37°C bath of water for thawing and culturing. DMEM was added, and the centrifugation of mixture was conducted for 5 min at 1500 rpm. The precipitate was put into a culture flask and placed in the CO_2_ incubator identically. After thawing, the cells were subcultured once.

### Morphologic Characterization of AdSCs

To confirm mesenchymal characteristics of AdSCs, the cells were assessed morphologically using an inverted microscope (Olympus, U.S.A.).

### Characterization of AdSCs by Reverse Transcriptase Polymerase Chain Reaction (RT-PCR)

The mesenchymal (CD90) and hematopoietic surface markers (CD34) were evaluated. To evaluate markers’ expression, RT-PCR was performed. Briefly, AdSCs of passage 4 were applied for total RNA isolation using a column RNA isolation kit (Denazist-Asia, Iran). Total concentration of RNA was evaluated by spectrophotometry. Synthesis of the complementary DNAs (cDNAs) by AccuPower® CycleScript RT PreMix (Bioneer, Korea) was conducted on RNA strains as a template. A volume of 20 µl, including 15 µl RNA with diethylpyrocarbonate-treated water (DEPC-water) was applied in every reaction. Thermal cycles (n=12) were conducted, including cDNA synthesis (4 min at 42°C), melting secondary structure and cDNA synthesis (30 sec at 55°C), primer annealing (30 sec at 20°C), and inactivation (5 min at 95°C). Then, template (cDNA) 1 µl was blended with other reagents such as taq DNA polymerase, PCR buffer, H_2_O, MgCl_2_, dNTPs, and forward, CD34 and CD90 reverse primers. Microtubes with the mixture 20 µl were transferred to a thermocycler (Eppendorf, Germany). Cycles of amplification (n=30) were conducted, including denaturation at the beginning (5 min at 95°C), and continued by 30 cycles of denaturation (30 sec at 95°C), annealing (30 sec 64°C, 62°C, or 61°C) and extension (30 sec at 72°C), and final polymerization (5 min at 72°C). Subsequently, using 1.5% agarose gel electrophoresis nearby an appropriate suitable DNA size marker, PCR products were evaluated and then stained and visualized through UV light.

### Adipogenic and Osteogenic Characterization of AdSCs

Passage four cells were applied, and adipogenic and osteogenic differentiations potential of AdSCs was determined. AdSCs were cultured in 6-well plates. After 70% confluency, the cells were grown 3 weeks in adipogenic or osteogenic media. Osteogenic medium consisted of supplement of low glucose DMEM with 10% FBS (Sigma-Aldrich), 10 mM b-glycerophosphate (Sigma-Aldrich), 0.05 mM ascorbate-2-phosphate (Wako Chemicals, Richmond, VA, U.S.A.), 100 nM dexamethasone (Sigma-Aldrich), and 1% antibiotic/antimycotic (Sigma-Aldrich). Furthermore, adipogenic medium consisted of low glucose DMEM with 10% FBS, 0.5 mM isobutyl-methylxanthine, 200 μM indomethacin, 10 μM insulin, and 1 μM dexamethasone (Sigma-Aldrich). Every 3 days, the adipogenic or osteogenic media was changed. Three weeks later, the cells were fixed in 10% formalin solution (Sigma-Aldrich) for 10 min. To identify osteogenic differentiation and calcified extracellular matrix, using Alizarin Red (Sigma-Aldrich), cells were stained, and then washed subsequently two times using distilled water. Adipogenic differentiation was confirmed by observing lipid droplets via staining with Oil Red O (Sigma-Aldrich).

### Transplantation of AdSCs

After 35 days of the final busulfan injection, the guinea pigs were anesthetized by ketamine (40 mg/kg, Woerden, Netherlands) and xylazine (0.5 mg/kg Alfazyne®, 2%, Woerden, Netherlands). They were transferred into dorsal recumbency, and after the abdominal area disinfection, 1 cm incision was conducted at the midline of the abdomen to reach the peritoneal cavity. The attached fat pad in the right seminiferous, and testis was gently pulled using an iris forceps until the testis was removed and observed under a microscope (Zeiss OPMI operating microscope, Carl Zeiss Meditec, Jena, Germany). A glass pipette was pulled and attached to the tube. To inject AdSCs into testis, they were labeled by sterile trypan blue (1:1, v/v), and then loaded to the polyethylene tube connected to a 1 ml syringe as a sign to track the injection. The syringe was pressed to force the suspension of cell to the pipette gently. A stereomicroscope was used to explore the seminiferous tubules, while 100 µl AdSCs mixture containing 10^6^ cells was administered into the lumen of busulfan-treated testis tubules. The testis was then transferred to the abdominal cavity, and the abdominal muscles and skin were closed by sutures. The left testis was regarded as control.

### Histopathologic and Histomorphometric Assessment

Guinea pigs testes were tested microscopically to detect any spontaneous spermatogenesis. The spermatogenesis duration in guinea pig is approximately 34 days having 4 cycles lasting 8.5 days [26]. It is also found that the primary spermatogenesis wave has duration between 40-45 days in animals [27]. The animals were euthanized after 60 days (about seven cycles) following transplantation of cell by ether. Then, both testes were dissected out and kept in 10% formalin buffer. From the equatorial and polar regions, five vertical sections were provided histologically with a thickness of 5 µm and stained via hematoxylin-eosin and evaluated using light microscope to detect any spermatogenesis changes.

The tubules were assessed for any spermatocytes, spermatogonia, and spermatids. In tubules, 10 similar circular transverse sections were undertaken, each was placed in a different testis area, employing a

systematic random protocol to assess the stereological indices as reported before [13]. The average diameter of seminiferous tubule (d) was examined through determining the mean of two diameters at right angles (D_1_ and D_2_). The tubules’ cross-sectional area (A_c_) was calculated using equation of A_c_=πD^2^/4 where π is 3.142, and D as mean diameter of the seminiferous tubules [13].

Rating testis for spermatogenesis potential was measured using a modified spermatogenesis indices on 0 to 6 scale [13].

The index depended upon presence of spermatogenesis cells in the testicular tissue, the count of included cell layers, types of cells and appearance of late spermatids. The criteria and index were as follows in each tubule: no spermatogenesis cells were scored as 0; only spermatogonia was scored as 1; detection of spermatogonia and spermatocytes without spermatids was scored as 2;

observation of spermatogonia, spermatocytes, and <50 round spermatids were scored as 3; spermatogonia, spermatocytes, and round spermatids up to 50-100 were scored as 4; spermatogonia, spermatocytes, and round spermatids up to 100-150 were scored as 5; and all cell types and >150 late spermatids in each tubule were scored as 6 [13].

### Statistical Analysis

The obtained data were statistically analyzed by one-way ANOVA. The LSD post-hoc test was applied to compare data using SPSS (Version 18, Chicago, IL, U.S.A.). Mann-Whitney U-test was used to evaluate the seminiferous tubules’ spermatogenesis index. P-values less than 0.05 were considered significant.

## Results

### Morphology of AdSCs

**Figure-1:**
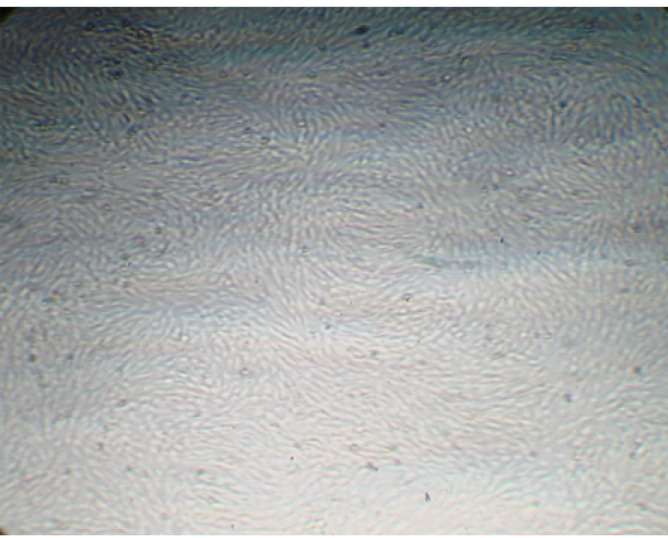
Guinea pig adipose tissue-derived stem cells (AdSCs) during the fourth passage (×40)

AdSCs demonstrated spindle-shaped morphology and in all passages were connected to plastic culture flasks (Figure-1).

### Characterization of AdSCs by RT-PCR

**Figure-2:**
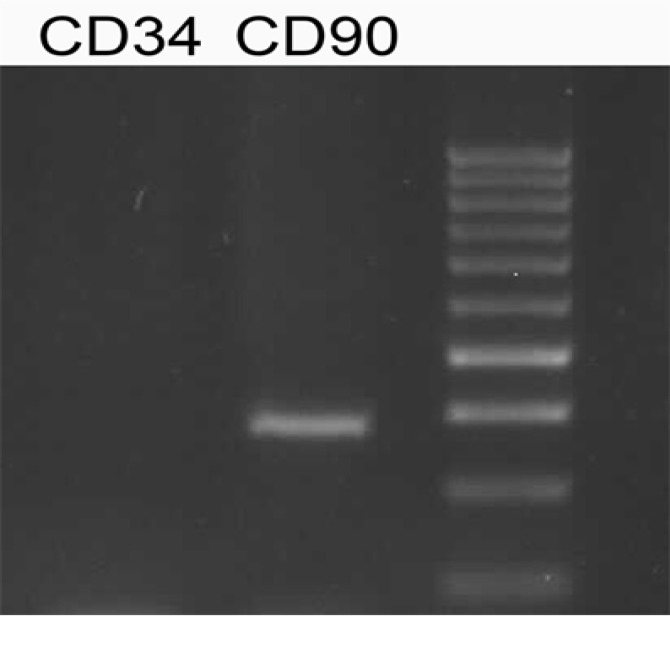
Presence of mesenchymal markers (CD90, 281bp) and absence of hematopoietic marker (CD34, 484bp) in guinea pigs adipose tissue-derived stem cells

AdSCs were found positive for CD90 expressions as MSC markers and negative for CD34 as a hematopoietic cell marker (Figure-2).

### Adipogenic and Osteogenic Differentiation of AdSCs

**Figure-3:**
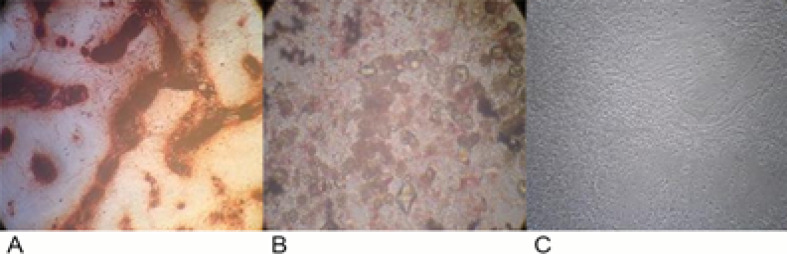
Adipose tissue-derived stem cells in guinea pig cultivated in **(A)** osteogenic medium and stained with Alizarin Red (×100); **(B)** in adipogenic medium stained with Oil Red O at day 21 after induction (×200); and **(C)** in culture media without differentiation medium as control (×100)

AdSCs in adipogenic and osteogenic differentiation media were differentiated into osteoblasts and adipocytes, respectively. After fixation and staining, adipogenic induction was verified by observation of lipid droplets, and in osteogenic medium, calcium deposits were visualized by confirming osteogenic differentiation of AdSCs (Figure-3).

### Histologic Findings

**Figure-4:**
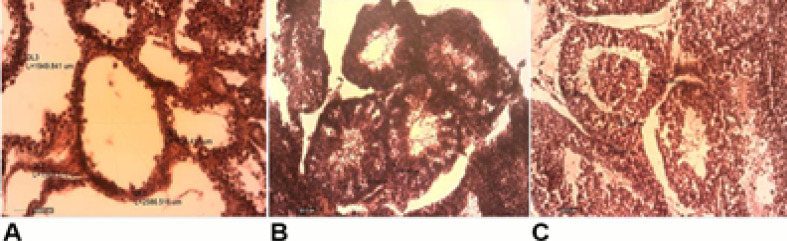
**A**: Sections of seminiferous tubules of busulfan-treated animals. The seminiferous tubules lack any germinal cell layer indicating the absence of spermatogenesis; **(B, C)** After transplantation of AdSCs into seminiferous tubules, most of tubules appeared to have spermatogenic cells (×100, hematoxylin and eosin staining) (White line indices are equal to 100 μm)

After double injections of busulfan to induce azoospermia in guinea pigs, depletion of germ cells was noted and only Sertoli cells were visible (Figure-4). In the busulfan-treated group, after 60 days, degenerative variations, including germinal epithelium’s degeneration and seminiferous tubules’ atrophy were observed. In seminiferous tubules, enlargement and vacuolation of lumen were seen, while germinal epithelium was atrophic and the seminiferous tubules’ peripheral zone resembled thin bands. No spermatozoa were also seen in busulfan-treated group’s epididymis (Figure-4A).

When transplantation of AdSCs was undertaken, spermatogonia appeared in seminiferous tubules (Figure-4B). The tubules were full of germinal cells, including primary spermatocytes, spermatids, spermatogonia, and spermatozoa. Most epididymis tubules in the cell-transplanted group revealed spermatozoa, even some of the tubes were vacant. Spermatozoa were detected in epididymis of AdSCs-treated group, while normal intact guinea pigs showed greater condensed germinal epithelium. All epididymis were full of spermatozoa.

### Histomorphometric Findings

**Figure-5:**
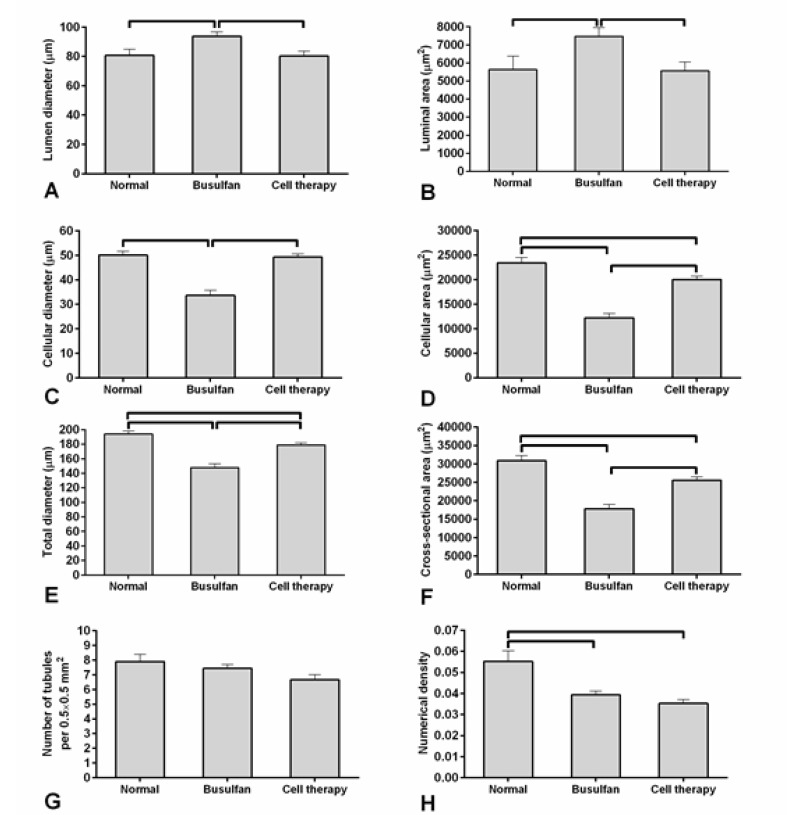
The mean (standard error) of stereological indices of seminiferous tubules in busulfan-induced azoospermic testis treated with adipose tissue-derived stem cells in comparison with busulfan-treated and control groups in guinea pig. **A)** Lumen diameter (μm), **B)** Luminal area (μm2), **C)** Cellular diameter (μm), **D)** Cellular area (μm2), **E)** Total diameters (μm), **F)** Cross sectional area of the tubule (μm2). **G)** Number of tubules (mm2), **H)** Numerical density. The lines above the columns show the statistical difference between the groups (P-value<0.05).

Histomorphometric evaluations showed that there were significant variations for luminal area and luminal diameter of testes’ seminiferous tubules between busulfan and AdSCs-treated experiment groups (P<0.05), however the AdSCs-treated group was identical to the control group (P<0.05, Figure-5A and Figure-5B). Furthermore, the cellular diameter, cellular area, total diameter and cross-sectional area of seminiferous tubules in the AdSCs transplanted and the control groups were higher than the busulfan-treated group (P<0.05, Figure-5C, 5D, 5E and 5F).

**Figure-6:**
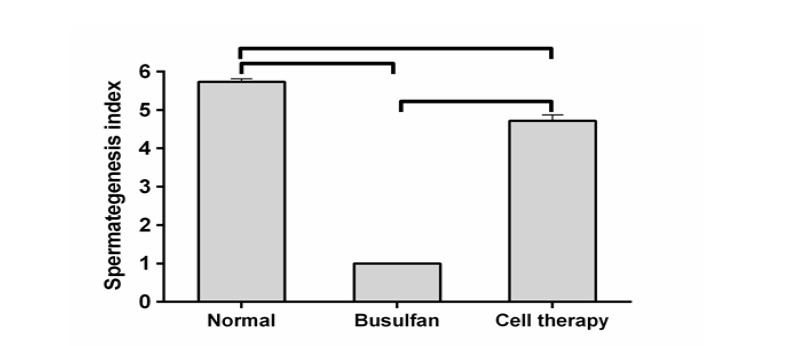
Mean and standard error of spermatogenesis index of seminiferous tubules in busulfan-treated, cell transplantation (therapy) and control (normal) groups of guinea pig. Spermatogenesis index scores: 0, no spermatogenesis cells; 1, only spermatogonia; 2, detection of spermatogonia and spermatocytes without spermatids; 3, observation of spermatogonia, spermatocytes, and <50 round spermatids; 4, spermatogonia, spermatocytes, and round spermatids up to 50-100; 5, spermatogonia, spermatocytes, and round spermatids up to 100-150; 6, all cell types and >150 late spermatids in each tubule. The lines above the columns indicate a significant difference between the groups (P-value0.05).

The number of seminiferous tubules in every unit area related to the cell-transplanted group was the same as that of busulfan-treated and control groups (P>0.05, Figure-5G). The numerical density of seminiferous tubules in the cell-transplanted group was the same as the busulfan-treated testes, but both of them had differences with the control group (P<0.05, Figure-5H). Spermatogenesis index of the seminiferous tubules in control and cell-transplanted groups was greater than the busulfan-treated animals (P<0.05, Figure-6).

## Discussion

Busulfan is a treatment for myeloid leukemia and is also used before bone marrow transplantation [28]. The drug is an alkylating agent inhibiting mitosis in G1 phase [29], inducing apoptosis and destroying mitotic cells, including germ cells, resulting in infertility and azoospermia [13].

Cell transplantation was shown as a new approach to treat disorders such as azoospermia [22].To cure non-obstructive azoospermia, hormonal therapies or surgical interventions were demonstrated with low efficacy [30]. In our study, AdSCs were used in treating azoospermic guinea pigs. Spindle shape and fibroblast-like morphology of the cells was observed, while all cells were adherent to plastic culture flasks. RT-PCR findings and adipogenic and osteogenic differentiation properties of AdSCs confirmed mesenchymal nature of isolated cells [20, 21].We showed that transplantation of AdSCs in azoospermic guinea pigs could successfully recover fertility and induce spermatogenesis. Similar findings demonstrated that AdSCs could recover fertility in the busulfan-treated azoospermia in rats [23, 31], hamsters [14], and mice [32]. Cakici *et al*. [31] reported that AdSCs transplantation following 12 weeks could recover busulfan-treated infertile male rats and induce spermatogenesis. Lue *et al*. [32]

showed that transplanted AdSCs in the model of busulfan-treated infertile mouse may differentiate into Sertoli, germ, and/or Leydig cells. Histomorphometric findings were different from those obtained by Tamadon *et al*. [14],

but identical to those obtained by Rahmanifar *et al*. [22]. The variation may be due to the difference in sample size, type of transplanted cells, and azoospermic animal models affecting the anatomical differences in the reproductive system. The luminar area and diameter of seminiferous tubules receiving stem cells were lower those of azoospermic animals and equal to those of normal guinea pig, explaining the difference in the results. Before cell transplantation, an increase in luminal area was observed in the azoospermic group, and a decline in cellular layers led to a decline in tubal resistance and a collapse in tubules under intra-tubular hydrostatic pressure. Intra-tubular hydrostatic pressure decrease may be the cause of the increase of the cellular layer diameter in AdSCs-treated tubes [14, 22].

Several studies evaluated the MSCs’ differentiation potential into spermatozoa in rats and mice regarding intra-seminiferous tubule [33, 34] and intra-testicular injection of bone marrow-derived MSCs (BMSCs) [35].

Monsefi *et al*. [35] showed that testis in infertile rats received BMSCs, the transplanted cells may differentiate in seminiferous tubules into germ cells. The MSCs’ differentiation potential into spermatozoa has been found in mice [32], rats [23, 34, 36] and hamsters [14]. Cakici *et al*. [31] reported that differentiation did not occur in all tubules [23]. In addition, in vitro studies for BMSCs confirmed their in-vitro differentiation into germ cells [37, 38].Sperm differentiation of umbilical cord MSCs of human transplanted into seminiferous tubule of immunodeficient mouse has been demonstrated [39]. It was shown that cell transplantation in azoospermic animals could depend on species [14]. Seminiferous tubules regulate spermatogenesis and Sertoli cells as immune-privileged for successful cell transplantation without the need for chronic immunosuppression therapy. The immunoprotective nature of Sertoli cells has been presented in another study [40]. Sertoli cells make the microenvironment suitable for differentiation and proliferation of spermatogonial cells. Our findings confirmed that AdSCs could reconstitute tubular microenvironment, providing the chance for other germinal cells inactivated to proliferate in the host seminiferous tubules [14]. There is evidence of the MSCs’ hypoimmunogenic nature, therefore there are implications for allogeneic therapy [14, 41]. Intravenous injection of BMSCs had immunosuppressive effects on production of antisperm antibody in allogeneic transfusion after testis rupture in mice [14, 42]. Immunological tolerance in Sertoli cells [40] can protect allotransplanted cells post-transplantation. Other points were also noticed in allotransplantation of stem cells in seminiferous tubules: (i) the immune privilege character of seminiferous tubules. Certain sites of some tissues in immunocompetent wild type mammalians are immune privilege such as the eye, brain, uterus which is pregnant and the testes [43], (ii) busulfan’s pharmacologic property as a chemotherapeutic agent frequently applied in lower doses for a long time to cure chronic myeloid leukemia [44] and (iii) prior to any hematopoietic cells’ allotransplantation [45].

Thus, allotransplantation of MSCs was assumed as a method to treat azoospermia cell.

## Conclusion

Our finding indicated the recovery of pathological variations in seminiferous tubules following transplantation of cell. The transplanted AdSCs could induce production of all germinal cell types in testicular seminiferous tubules, and were an effective source to treat azoospermia. Therefore, AdSCs can be a proper candidate for azoospermia improvement and infertility recovery.
